# Characteristics of siRNAs derived from *Southern rice black-streaked dwarf virus* in infected rice and their potential role in host gene regulation

**DOI:** 10.1186/s12985-017-0699-3

**Published:** 2017-02-10

**Authors:** Donglin Xu, Guohui Zhou

**Affiliations:** Key Laboratory of Microbial Signals and Disease Control of Guangdong Province, College of Agriculture, South China Agricultural University, 510642 Guangzhou, Guangdong China

**Keywords:** *Southern rice black-streaked dwarf virus*, Virus-derived siRNAs, RNA silencing, Deep sequencing, RT-qPCR, Host defense, Target genes

## Abstract

**Background:**

Virus-derived siRNAs (vsiRNAs)-mediated RNA silencing plays important roles in interaction between plant viruses and their hosts. *Southern rice black-streaked dwarf virus* (SRBSDV) is a newly emerged devastating rice reovirus with ten dsRNA genomic segments. The characteristics of SRBSDV-derived siRNAs and their biological implications in SRBSDV-rice interaction remain unexplored.

**Methods:**

VsiRNAs profiling from SRBSDV-infected rice samples was done via small RNA deep sequencing. The putative rice targets of abundantly expressed vsiRNAs were bioinformatically predicted and subjected to functional annotation. Differential expression analysis of rice targets and RNA silencing components between infected and healthy samples was done using RT-qPCR.

**Results:**

The vsiRNA was barely detectable at 14 days post infection (dpi) but abundantly present along with elevated expression level of the viral genome at 28 dpi. From the 28-dpi sample, 70,878 reads of 18 ~ 30-nt vsiRNAs were recognized (which mostly were 21-nt and 22-nt), covering 75 ~ 91% of the length of the ten genomic segments respectively. 86% of the vsiRNAs had a <50% GC content and 79% of them were 5’-uridylated or adenylated. The production of vsiRNAs had no strand polarity but varied among segment origins. Each segment had a few hotspot regions where vsiRNAs of high abundance were produced. 151 most abundant vsiRNAs were predicted to target 844 rice genes, including several types of host resistance or pathogenesis related genes encoding F-box/LRR proteins, receptor-like protein kinases, universal stress proteins, tobamovirus multiplication proteins, and RNA silencing components OsDCL2a and OsAGO17 respectively, some of which showed down regulation in infected plants in RT-qPCR. GO and KEGG classification showed that a majority of the predicted targets were related to cell parts and cellular processes and involved in carbohydrate metabolism, translation, and signal transduction. The silencing component genes *OsDCL2a*, *OsDCL2b*, *OsDCL4*, and *OsAGO18* were down regulated, while *OsAGO1d*, *OsAGO2*, *OsRDR1* and *OsRDR6* were up regulated, significantly, upon SRBSDV infection.

**Conclusions:**

SRBSDV can regulate the expression of rice RNA silencing pathway components and the virus might compromise host defense and influence host pathogenesis via siRNA pathways.

**Electronic supplementary material:**

The online version of this article (doi:10.1186/s12985-017-0699-3) contains supplementary material, which is available to authorized users.

## Background

RNA silencing or RNA interfering (RNAi) is an evolutionarily conserved gene inactivation mechanism that universally exists in eukaryotic organisms [1, 2] and plays critical roles in developmental regulation, response to stresses, and host defense against transposons and viruses [3–5]. In plants, RNA silencing is triggered by double-stranded (ds) or highly structured single-stranded (ss) RNAs, which are processed by Dicer-like (DCL) ribonucleases into two classes of small RNA molecules - microRNAs (miRNAs) sized about 21 nucleotides (nt) and small interfering RNAs (siRNAs) of 21 ~ 24 nt [6]. Invasion of plant viruses may initiate DCL-mediated biogenesis of primary virus-derived siRNAs (vsiRNAs) from double-stranded viral replicative intermediates (RI) or secondary-structured ssRNA genomic regions [7–9], and then the vsiRNAs are incorporated into Argonaute (AGO) proteins to form the RNA-induced silencing complex (RISC) that cleaves the viral RNAs or host mRNAs in a sequence-specific manner [9–13]. The RNA silencing mechanism can be considered as a type of pathogen-associated molecular pattern (PAMP)-triggered immunity (PTI) [5]. RNA silencing effects can be amplified by host RNA-dependent RNA polymerases (RDR), which are acquired to convert aberrant RNAs (including the RISC-cleaved products) into dsRNAs, the materials for biogenesis of secondary vsiRNAs directed by DCLs [14, 15]. For survival, plant viruses have evolved various viral suppressors of RNA silencing (VSRs) to overcome RNA silencing-mediated defense [5, 16]. For the counter-counter defense, plants constantly evolve disease resistance (*R*) genes which encode R proteins to interact with VSR, triggering effector-triggered immunity (ETI) and leading to hypersensitive response (HR)-based resistance [5, 17].

Many DCLs, AGOs and RDRs contribute to RNA silencing-based plant antiviral defense. DCL2 and DCL4 process dsRNA molecules into 22- and 21-nt vsiRNAs in a hierarchical and redundant manner respectively, which play essential roles in resistance to positive-strand RNA viruses, while DCL3 mainly functions to produce 24-nt vsiRNAs involved in plant resistance to DNA viruses [5, 14, 18, 19]. It has been demonstrated that specific AGOs preferentially bind different small RNAs dependent on their 5’-terminal nucleotides [5, 11, 20–22]. RDR1, RDR2 and RDR6 are the major effectors involved in defense of *Arabidopsis thaliana* specifically against various viruses [14, 15, 17, 19]. In rice, DCL4 confers the defense against *Rice yellow mottle virus* (RYMV, genus *Sobemovirus*, unassigned family), which can be inhibited by the VSR, P1 protein of the virus [23]. AGO1 and AGO18 participate in the defense against *Rice stripe virus* (RSV, genus *Tenuivirus*, unassigned family) and *Rice dwarf phytoreovirus* (RDV, genus *Phytoreovirus*, family *Reoviridae*), wherein AGO1 is the direct antiviral effector, while AGO18, induced upon virus infection, combines with miR168 to sequester the down regulation of AGO1 by miR168 and thus confers a broad-spectrum antivirus resistance [24]. Also, OsRDR6 has been proven to play role in host resistance to RSV and RDV [25, 26]. Although vsiRNAs-endowed host defense against viruses is universal, they may benefit from the RNA silencing machinery in some scenarios. Recent research has discovered a role of vsiRNAs in targeting certain host genes, suggesting that vsiRNAs may modulate host response and symptomology through the RNA silencing machinery to facilitate virus survival and spread [12, 27–30].

*Southern rice black-streaked dwarf virus* (SRBSDV) is a newly emerged rice reovirus (genus *Fijivirus*, family *Reoviridae*) as well as an insect virus efficiently transmitted by the long-distance migratory pest, the white-backed planthopper (WBPH, *Sogatella furcifera*) in a persistent, circulative propagative manner [31, 32]. The virus has overspread to vast rice-growing areas in China and some eastern Asian countries in recent years and caused severe loss to rice production there [31, 32]. The virus has a genome consisting of ten dsRNA segments named S1 to S10 according to their molecular weights from large to small. SRBSDV S1 ~ S4, S6, S8 and S10 each encodes one protein, whereas S5, S7 and S9 are bicistronic. SRBSDV P1 ~ P4, P8 and P10 are structural proteins and the other seven proteins are nonstructural [31]. Sequence analysis suggested that its P1 ~ P4 are the viral RNA-dependent RNA polymerase (RdRP), major core structural protein, outer-shell B-spike protein and capping enzyme, respectively [31, 33, 34]. SRBSDV P6 is a VSR, and it has complicated interactions with P5-1 and P9-1 to form viroplasm matrices in WBPH cells [35–38]. The P7-1 was found to form tubule structures in WBPH cells, which may serve for intercellular virus transportation and spread in the insect vector [39, 40]. The P9-1 is the major component of viral viroplasm that is important to viral replication in insect vectors [41]. The P8 and P10 are putative minor core protein and major outer capsid protein, respectively [31, 33, 34]. Previous studies revealed that SRBSDV infection may regulate the expression of a bunch of rice miRNAs [42] and some of the RNAi-related genes in WBPH under different temporal conditions, suggesting interactions of the virus and host silencing pathways [43]. It has been demonstrated that the core component Dicer-2 of siRNA pathway has decisive influences on SRBSDV accumulation and dissemination in insect and vector competence [44]. Most recently, SRBSDV-derived siRNAs were reported to originate equally from both strand orientations of the viral genome by the Dicer enzyme in viruliferous WBPH, however, no further information about the vsiRNAs was presented by the authors [45]. To better understand SRBSDV-derived siRNAs and gain insight into their biological implications, in this study we characterized the vsiRNAs present in infected rice by deep sequencing and explored their potential role in host gene regulation, and the influence of SRBSDV infection on rice RNA silencing pathway was analyzed as well.

## Methods

### Plant cultivation and virus inoculation

Seedlings of rice (*Sativa japonica* L. cv. Nipponbare) used in this study were obtained using the water planting method described by Yoshida et al. [46]. Briefly, rice seeds after soaked in water for 24 h were germinated on wet towels at 30 °C and then sown in a 2-L beaker half-filled with culture solution to grow into three- to four-leaf stage seedlings in a growth chamber under the conditions of 25 ± 0.5 °C, RH 75 ± 5%, and 12 h light/12 h dark. Twenty seedlings with uniform growth were selected and each of them were inoculated with five WBPH nymphs in third- or fourth-instar, which previously were hatched and fed on the SRBSDV-infected rice plants maintained in our lab. In parallel, a group of seedlings were mock-inoculated with WBPH nymphs born and fed on healthy plants. The insects were allowed to feed on the seedlings for 48 h before being removed manually. Infection of each inoculated plant was confirmed by RT-PCR using the previous method [47] at 10 days post inoculation/infection (dpi).

### RNA extraction, small RNA sequencing and analysis of siRNAs

Leaf samples of ten SRBSDV-infected plants were harvested at 14 and 28 dpi, respectively, and pooled for total RNA extraction using a TRIzol Reagent (TaKaRa, Dalian, China). The obtained RNA products were treated by DNase I (TaKaRa) and their concentration, integrity and purity were verified by an Agilent Bioanalyzer 2000 system (Agilent, CA, USA). Deep sequencing of small RNAs was performed by Beijing Genome Institute (BGI, Shenzhen, China) using their Illumina HiSeq platform. In brief, sRNAs of 18 ~ 30 nt were recovered from a 15% denaturing polyacrylamide gel electrophoresis of total RNA, ligated with RNA adaptors, and reversely transcribed into cDNAs. These cDNAs were amplified by PCR and subjected to Solexa/Illumina sequencing. From the generated data, adapter sequences were trimmed and clean reads of small RNAs between 18 ~ 30 nt in length were extracted. These small RNA sequences were mapped to the SRBSDV genomic sequences (GenBank accession numbers: NC_014708 ~ NC_014717) and only those identical to viral sequences (of sense or antisense strands) within 2 nt mismatches were recognized as vsiRNAs. According to their abundance levels, single-nucleotide resolution maps of the vsiRNAs were drawn using the OriginPro 9.0 software to display the distribution hotspots along the viral genome.

### Prediction and annotation of target genes

Target genes prediction for the vsiRNAs of over 40 reads was perform by the online analysis tool psRNATarget (http://plantgrn.noble.org/psRNATarget/) [48] using default parameters and a rice transcript database, MSU Rice Genome Annotation (version 7). The predicted target genes were subject to BLAST alignment and Gene Ontology (GO) annotation using BLAST2GO software [49]. KEGG classification of the predicted target genes was done using KEGG Mapper - Annotate Sequence by BlastKOALA available on the Kyoto Encyclopedia of Genes and Genomes website (http://www.kegg.jp/kegg/tool/annotate_sequence.html).

### Reverse transcription-quantitative PCR

The total RNAs were extracted from the SRBSDV-infected (14 and 28 dpi) and mock-inoculated samples, treated by the DNase I, and used as templates for RT-qPCR, which was done using a One Step SYBR^®^ PrimeScript™ RT-PCR Kit II (Perfect Real Time) (TaKaRa) in a Thermal Cycler^®^ Dice Real Time System TP800 (TaKaRa) following the following the manufacturers’ instructions. The one-step amplification cycling conditions were 42 °C for 5 min, 95 °C for 10 s, and 40 cycles of 95 °C for 5 s and 60 °C for 30 s. After completion of qPCR cycling, the melting curves were generated at 95 °C to verify the specificity of amplification. For quantification of SRBSDV genomic segments (S1 and S10) and rice transcripts, a U6 small nuclear RNA gene and an actin gene of rice were used as internal control, respectively. All reactions were performed in triplicate. Data normalization based on Ct values was done by the 2^−△△Ct^ method [50] and *t*-test analysis was conducted using SPSS19.0 software. The RT-qPCR primers used in this study were listed in Additional file 1: Table S1.

## Results

### Profiling of SRBSDV-derived siRNAs in infected rice

**Fig. 1:**
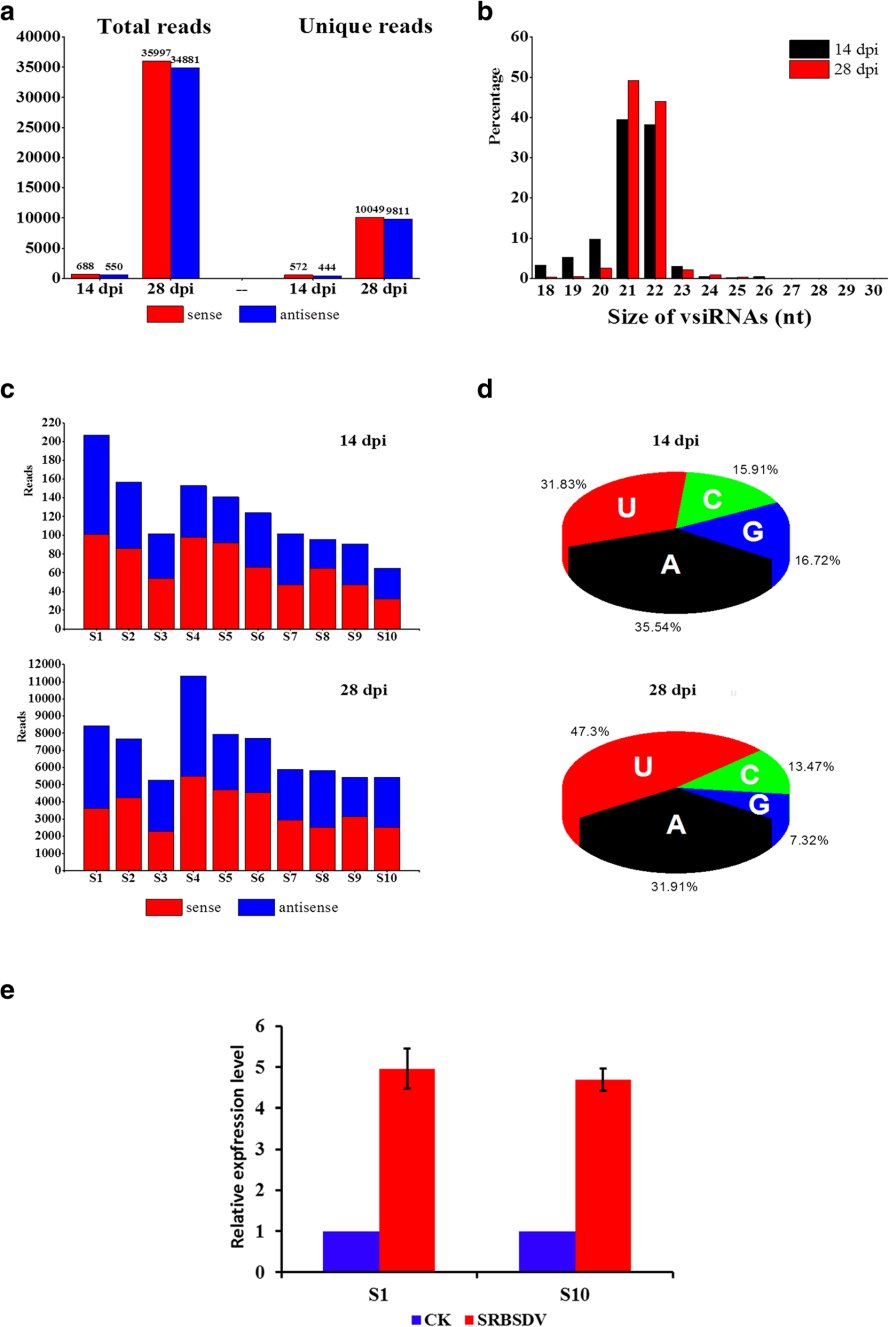
Identification of virus-derived siRNAs (vsiRNAs) from Southern rice black-streaked dwarf virus (SRBSDV)-infected samples at 14 and 28 dpi. **a** Amounts of total and unique vsiRNA reads from both strand orientations. **b** Size distribution of total reads. **c** Segment distribution of total reads. **d** Percentage frequencies of different 5’-terminal nucleotides in the identified vsiRNAs. **e** Elevated expression levels of SRBSDV genomic segments at 28 dpi in relative to at 14 dpi

A total of 10,479,093 and 12,131,156 clean reads were obtained from the small RNA libraries of SRBSDV-infected samples at 14 and 28 dpi, respectively. Among these, only 1,238 reads (0.01%, containing 1,016 unique sequences of 18 ~ 27 nt long) from the 14-dpi sample were mapped to the SRBSDV genome (36 sequences with 2 nt mismatch and the rest with perfect match), and the two most abundant sequences had only 23 and 11 reads, respectively (Additional file 2: Table S2). The GC content of these vsiRNAs varied from 16.7% to 66.7%, and 91% (927/1016) of the unique sequences had a GC content lower than 50%. In the 28-dpi sample, 70,878 reads (0.42%, containing 19,860 unique sequences of 18 ~ 30 nt long) were recognized as vsiRNAs with the most abundant one having 336 reads. Their GC contents were 20.8 ~ 73.7% and 86% (17141/19860) of the unique sequences had a GC content lower than 50% (Additional file 3: Table S3). There were 366 vsiRNAs present in both samples (Additional file 4: Table S4). More than one half of total vsiRNA reads (55.6%) and unique reads (56.3%) originated from sense strands of the viral genome at 14 dpi, whereas the amount (<51%) of sense strands-derived vsiRNAs was nearly equal to that of antisense strands-derived vsiRNAs (Fig. 1a). The molecules of 21- and 22-nt represented the majority of the recognized vsiRNAs in both samples - in total, 77.7% for 14 dpi and 93.3% for 28 dpi respectively (Fig. 1b). This suggests that OsDCL4 and OsDCL2 are the predominant enzymes for biogenesis of SRBSDV-derived siRNAs. The vsiRNA accumulation level varied among SRBSDV genomic segments from which they were generated (Fig. 1c). At 14 dpi, the greatest proportion (one sixth) of the vsiRNAs reads came from genomic S1, and one eighth from both S2 and S4, while those produced from S10 were the least abundant (5.3%). At 28 dpi, S4-derived vsiRNAs were the most abundant (representing 16% of the total reads), followed by those produced from S1 (11.9%), S5 (11.2%), S6 (10.9%) and S2 (10.2%), while S3 contributed the smallest part (7.4%).

The 5’ terminal nucleotides of the obtained vsiRNAs were examined and the result showed that two thirds of the vsiRNA reads generated at 14 dpi had an adenosine (A, 35.54%) or uridine (U, 31.83%) at their 5’ ends, and those having 5’ guanine (G) or cytosine (C) were less abundant; while in the 28 dpi sample, near half of the vsiRNAs had 5’ U, near one third were 5’-adenylated, and 5’ G was the least abundant (Fig. 1d). These results were similar to that from many RNA plant viruses [8] and suggested that SRBSDV-derived siRNAs mainly are recruited by the OsAGO1 and OsAGO2 to form the RISC, according to the AGO preferences for small RNAs revealed by the previous study [11].

Comparison of the abundance of SRBSDV S1 and S10 between the infected and uninfected samples by RT-qPCR analysis showed that the expression levels of the two segments at 28 dpi were 25 ~ 30 times (4.96 fold for S1 and 4.69 fold for S10) higher than that at 14 dpi respectively (Fig. 1e). This implied that the trace amount of vsiRNA accumulation at 14 dpi was due to limited titer of the virus in the early stage of infection. With regard to this, our following analyses are based on the vsiRNA data obtained from the 28-dpi sample.

### Distribution of vsiRNAs in the SRBSDV genome

**Fig. 2:**
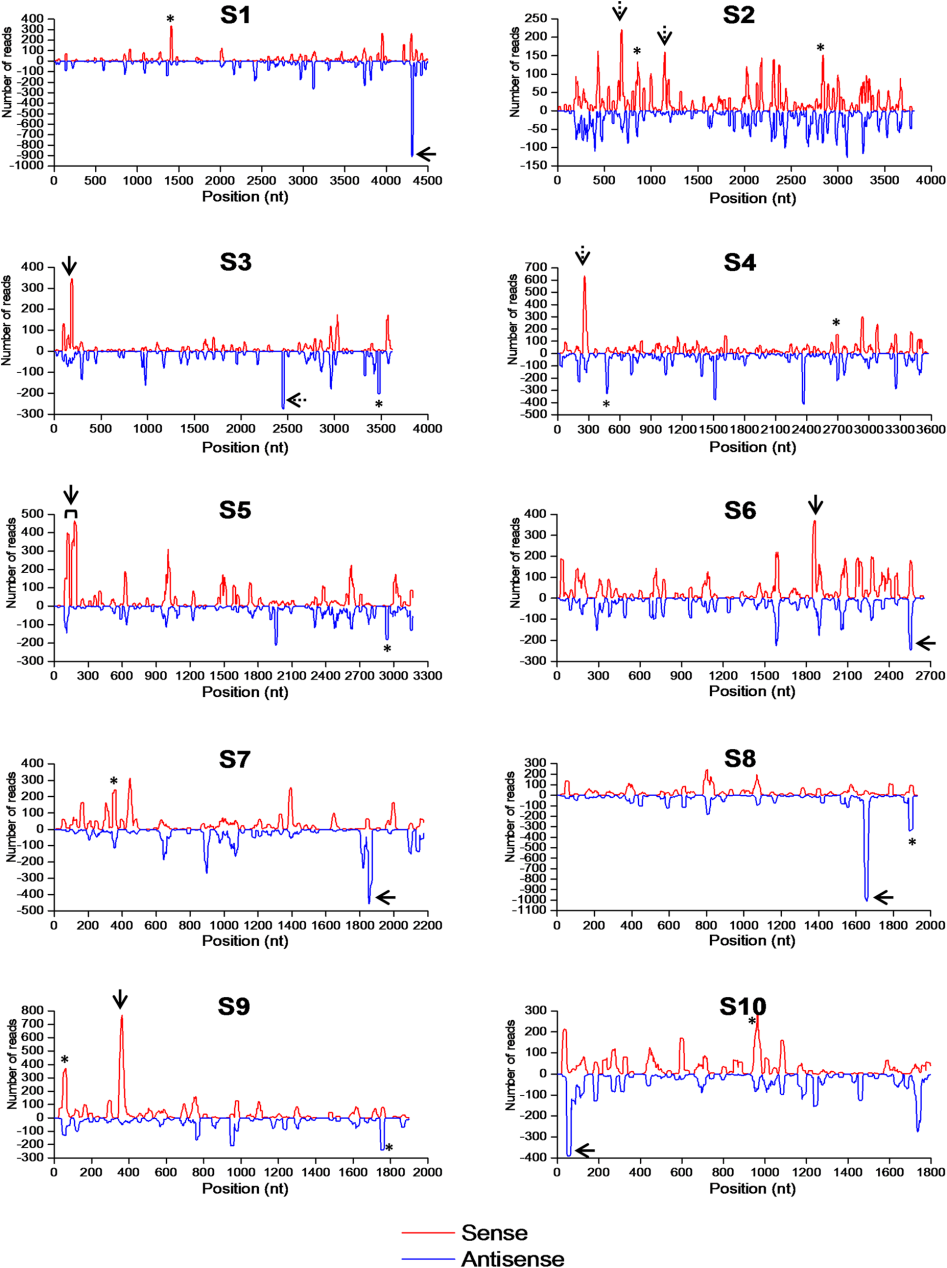
Single-nucleotide resolution maps of the virus-derived siRNAs (vsiRNAs) from the viral genomic segments S1~S10 at 28 dpi. The vsiRNA distribution hotspots are indicated by arrow signs. However, the hotspots marked by a dotted line arrow are not corresponding to the origins of the most abundant vsiRNAs in S2~S4. The solid-line arrow and asterisk signs indicate the origins of the two to three most abundant vsiRNAs in each segment

To gain further insight of the origin of the vsiRNAs, we calculated the frequency of the viral genome’s each single nt position for being covered by the vsiRNA reads (Additional file 5: Table S5), and drew single nucleotide resolution maps for each SRBSDV genomic segment according to the “nt position-frequency of being covered” data (Fig. 2). The results indicated that the vsiRNAs were not always continuously distributed through the genomic segments. Instead, there were 9.3% (S3) to 25.4% (S1) of nt positions not being covered by the vsiRNA sequences from both sense and antisense orientations (Additional file 5: Table S5). As shown in Fig. 2, distribution of vsiRNAs along the viral genome was highly heterogeneous. For each of S1 ~ S10, we defined the region as a distribution hotspot when it contained at least 18 consecutive nucleotides and all their single-nucleotide read counts reached two thirds of the highest value in the segment. It is showed that the S1(−), S7 (−), S8 (−) and S10 (−), S4 (+) and S9 (+) strands each contained one hotspot, both S2 (+) and S5 (+) strands contained two hotspots, and both sense and antisense strands of S3 and S6 had a hotspot respectively (Fig. 2). Except the one from the S6 (−) strand that was located in the 3’-UTR region, all the hotspots resided in coding regions of the viral genes. However, of these 14 hotspots, four peaks (from the S2 (+), S4 (+) and S3 (−) strands respectively) were not corresponding to the most abundant vsiRNA sequences identified from their respective segments; instead, some abundant vsiRNAs from several segments formed lower peaks in the single-nucleotide resolution maps (Fig. 2). These four exceptions came from the fact that the maps were created based on the frequencies of covering by vsiRNAs for single nt positions, not for complete siRNA-generating spots (i.e. 21 ~ 24 nt long regions). Four hotspots were found to generate a 21-nt and a 22-nt vsiRNA simultaneously: vsiRS1_1 and vsiRS1_2, vsiRS5_1 and vsiRS5_2, vsiRS5_5 and vsiRS5_6, and vsiRS6_1 and vsiRS6_2 from the same site of the S1 (−), S5 (+) and S6 (+) strands, respectively. The abundance levels of the 22-nt vsiRNAs were two times (S1) or slightly higher (S5 and S6) than that of the 21-nt ones (Table 1). This result indicated that the same sites on the viral genome may be recognized and cleaved by DCL4 and DCL2, and possibly, by DCL2 in preference. The similar phenomenon can also be found in other plant viruses [29, 30].

**Table 1:** The siRNAs of over 100 reads derived from Southern rice black-streaked dwarf virus

vsiRNA No.	Sequence (5’ to 3’)	Read count	Strand & position (nt)	GC%	Number of putative rice targets
vsiRS1_1	UAAUCGAGAUACACUCUGCGCC	336	(−) 4304-4325	50.0	2
vsiRS1_2	AAUCGAGAUACACUCUGCGCC	167	(−) 4304-4324	52.4	1
vsiRS1_3	UUGCGCAUUGUACUGACCUUGG	114	(+) 1400-1421	50.0	2
vsiRS1_4	UCGAGAUACACUCUGCGCCCA	104	(−) 4302-4322	57.1	1
vsiRS3_1	UUAGACGCAGAAUUGAAGAAUC	237	(+) 176-197	36.4	4
vsiRS3_2	UUCAUUAGGUACUUGAUCUGA	112	(−) 3466-3486	47.6	14
vsiRS4_1	UACAGGUAGUGAACACAAGCC	112	(+) 2684-2704	33.3	7
vsiRS4_2	UACGAGACAAUGCAAACUGUA	106	(−) 465-485	38.1	9
vsiRS5_1	ACACUCGUGACUCAAUUCUGCC	209	(+) 176-197	50.0	5
vsiRS5_2	CACUCGUGACUCAAUUCUGCC	176	(+) 177-197	52.4	5
vsiRS5_3	CACGAACAAGCAGACAACACU	162	(+) 112-132	47.6	2
vsiRS5_4	UAGCGUUGUGAUUGUGAUCAAA	128	(−) 2927-2948	36.4	7
vsiRS5_5	UUCACGACUUUGAAGACACCCA	125	(+) 155-176	45.5	0
vsiRS5_6	UUCACGACUUUGAAGACACCC	116	(+) 155-175	47.6	0
vsiRS6_1	UUGAGAGAACAAAGUGAUCGUU	122	(+) 1846-1867	36.4	9
vsiRS6_2	UGAGAGAACAAAGUGAUCGUU	119	(+) 1847-1867	38.1	8
vsiRS6_3	UGAGAUCUCUGUCCGUUAAAGA	119	(−) 2540-2561	40.9	14
vsiRS6_4	UGGGCCGACGUAGUUGAAAGAA	111	(+) 1579-1600	50.0	7
vsiRS7_1	UUGAAUAACAUUCUGUAGGAA	228	(−) 1851-1871	28.6	13
vsiRS7_2	UCCAAUCAAGAUUCAAGAGCU	109	(+) 344-364	38.1	7
vsiRS8_1	UCACUAGAAUCUACAUCGACCU	217	(−) 1882-1903	40.9	32
vsiRS8_2	UCGCAAUGGCACGAGUAGGACU	160	(−) 1648-1669	54.5	4
vsiRS8_3	AUGGCACGAGUAGGACUUAAUA	120	(−) 1643-1664	40.9	2
vsiRS8_4	AAUGGCACGAGUAGGACUUAAU	111	(−) 1644-1665	40.9	1
vsiRS9_1	AGAGAAUGGCAGACCUAGAGCG	159	(+) 47-68	54.5	0
vsiRS9_2	UACAGUACCUCCAUUGAACACU	147	(−) 1746-1767	40.9	13
vsiRS9_3	ACAGAUCAAUUGGACUUGGCU	143	(+) 349-369	42.9	8
vsiRS10_1	UUAUGGAUAAGAUCGGGCGCUA	134	(−) 44-65	45.5	3

### Prediction of putative rice target genes of vsiRNAs

In this study, we used the small RNA target analysis server psRNATarget [48] to explore the rice genes putatively targeted by SRBSDV-derived siRNAs. Because of the large number of vsiRNA species identified, only the 168 vsiRNAs with over 40 reads were selected for target prediction, including seven sequences of 40 ~ 58 reads from S2, the only segment not producing vsiRNAs of over 100 reads (Table 1). In total, 975 putative pairs of vsiRNA-target were predicted, including 151 unique vsiRNAs and 844 individual rice genes (Additional file 6: Table S6). Twenty-five out of the 28 most abundant siRNAs from nine segments (excluding S2) were predicted to each target 1 ~ 32 host genes, respectively, while three of them (vsiRS5_5, vsiRS5_6 and vsiRS9_1) had no rice targets (Table 1). Notably, a vsiRNA coming from 3’-UTR region of S8 putatively had 32 targets, 26 of which were retrotransposons (Fig. 2; marked by an asterisk). Besides, the seven most abundant vsiRNAs from S2 were predicted to have 1 ~ 6 rice targets, respectively. Annotation of the 844 genes predicted was done referring to the rice transcript database MSU Rice Genome Annotation (version 7). The result showed that a number of annotated targets were related to disease/stress response, including 33 *R* genes (13 of which were annotated as NBS-LRR or NBS protein genes), 17 F-box/LRR or F-box domain-containing protein genes, 15 receptor-like protein kinase (RLK) genes, five universal stress protein (USP) genes, and six tobamovirus multiplication protein genes (*TOM*). A majority of plant *R* genes encode NBS-LRR proteins that play roles in pathogen sensing/detection and host defense against a variety of pathogens including bacteria, fungi, nematodes and viruses [51–53], and some *R* genes may interact with VSRs to induce HR-mediated resistance [5, 54, 55]. Many plant F-box proteins as the subunits of the SCF complex, a type of ubiquitin E3 ligase, are involved in multiple biological and developmental processes and stress responses [56, 57] and can be regulated by the RNA silencing pathways in plants [58]. Also, it has been reported that F-box proteins can reduce the expression of AGO1 at protein level expression in *Arabidopsis*, and some plant viruses encode an F-box domain-containing VSR to degrade this antiviral component through the autophagy pathway [59, 60]. RLKs are a large family of proteins that function in various signal transduction pathways and participate in hormonal response pathways, cell differentiation, plant growth and development, self-incompatibility, and pathogen recognition and resistance [61–63]. USPs can increase drought tolerance of plants [64, 65]. TOM proteins are a type of plant transmembrane proteins that are required by tobamoviral genome replication and may suppress the host’s RNA silencing process when they are overexpressed [66]. Our analysis suggested that the several kinds of stress resistance genes above-mentioned were likely down regulated by vsiRNAs, making the SRBSDV-infected host become more susceptible to other biotic or abiotic stimuli. Besides, two rice genes LOC_Os03g38740.1 and LOC_Os02g07310.1, putatively targeted by the vsiRS8_16 and vsiRS2_1, were annotated as *OsDCL2a* and *OsAGO17*, respectively (Additional file 6: Table S6). *OsAGO17* is male gametophyte-specific and likely involved in rice pollen development regulated by miRNAs [67]. Moreover, seven chloroplast envelope membrane protein or chloroplast precursor-related genes were annotated, implying that photosynthesis pathways might be influenced by vsiRNAs in infected rice.

**Fig. 3:**
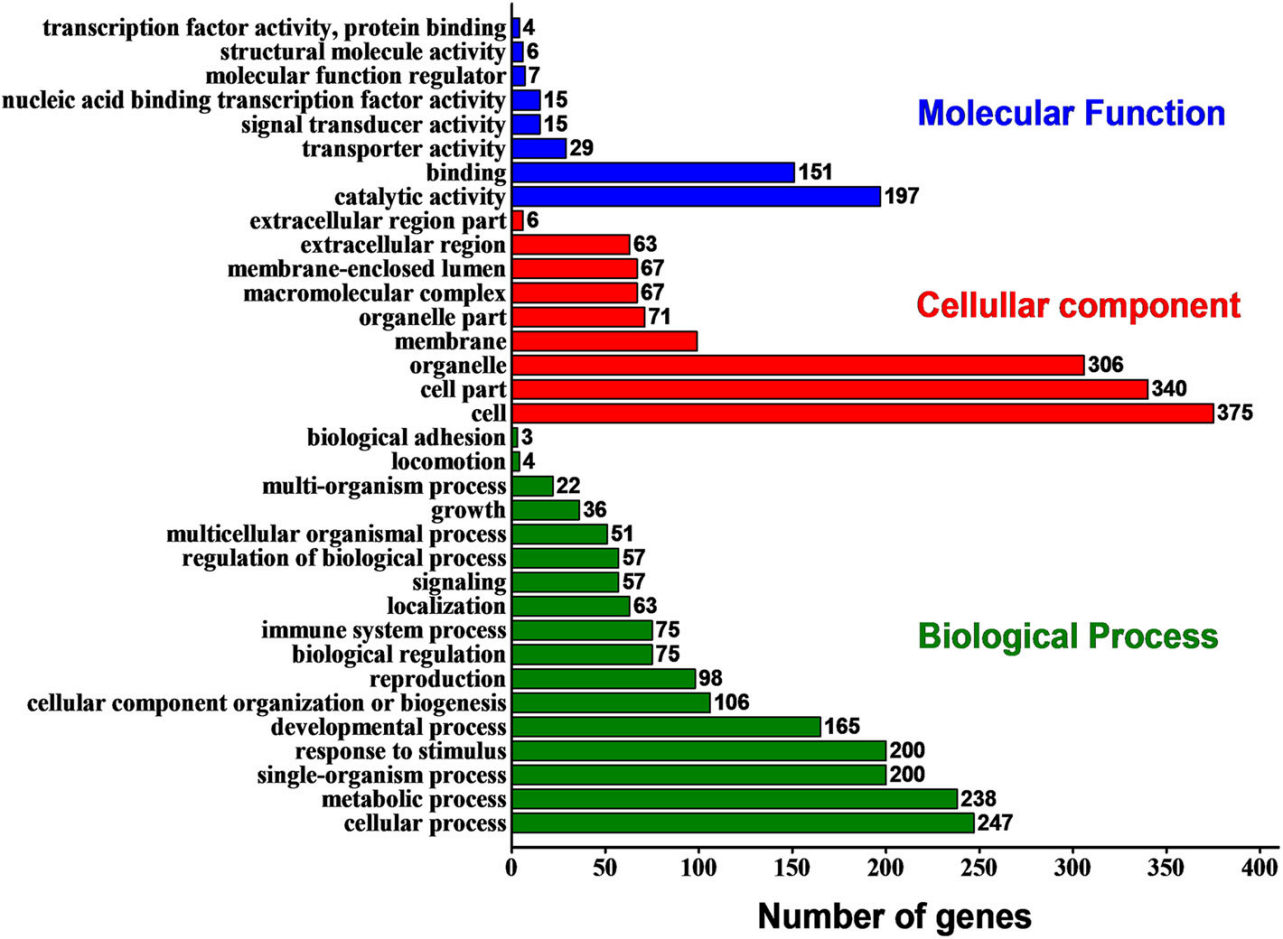
GO classification of predicted rice target genes of Southern rice black-streaked dwarf virus-derived siRNAs

In BLAST2GO analysis, a total of 409 predicted target genes of vsiRNAs were annotated and classified into three GO categories (biological process, cellular component, and molecular function with 366, 378, 294 individual genes respectively) and 34 subcategories (Fig. 3). The three most highly represented GO terms were “cell”, “cell part” and “organelle” under the cellular component category, comprising 75 ~ 92% of the annotated genes; five terms under the biological process section, “cellular process”, “metabolic process”, “single-organism process”, “response to stimuli” and “developmental process”, represented 40 ~ 60% of the classified targets; and the two most abundant GO terms under the molecular function category, “catalytic activity” and “binding”, included 48% and 37% of the annotated genes respectively (Fig. 3).

**Fig. 4:**
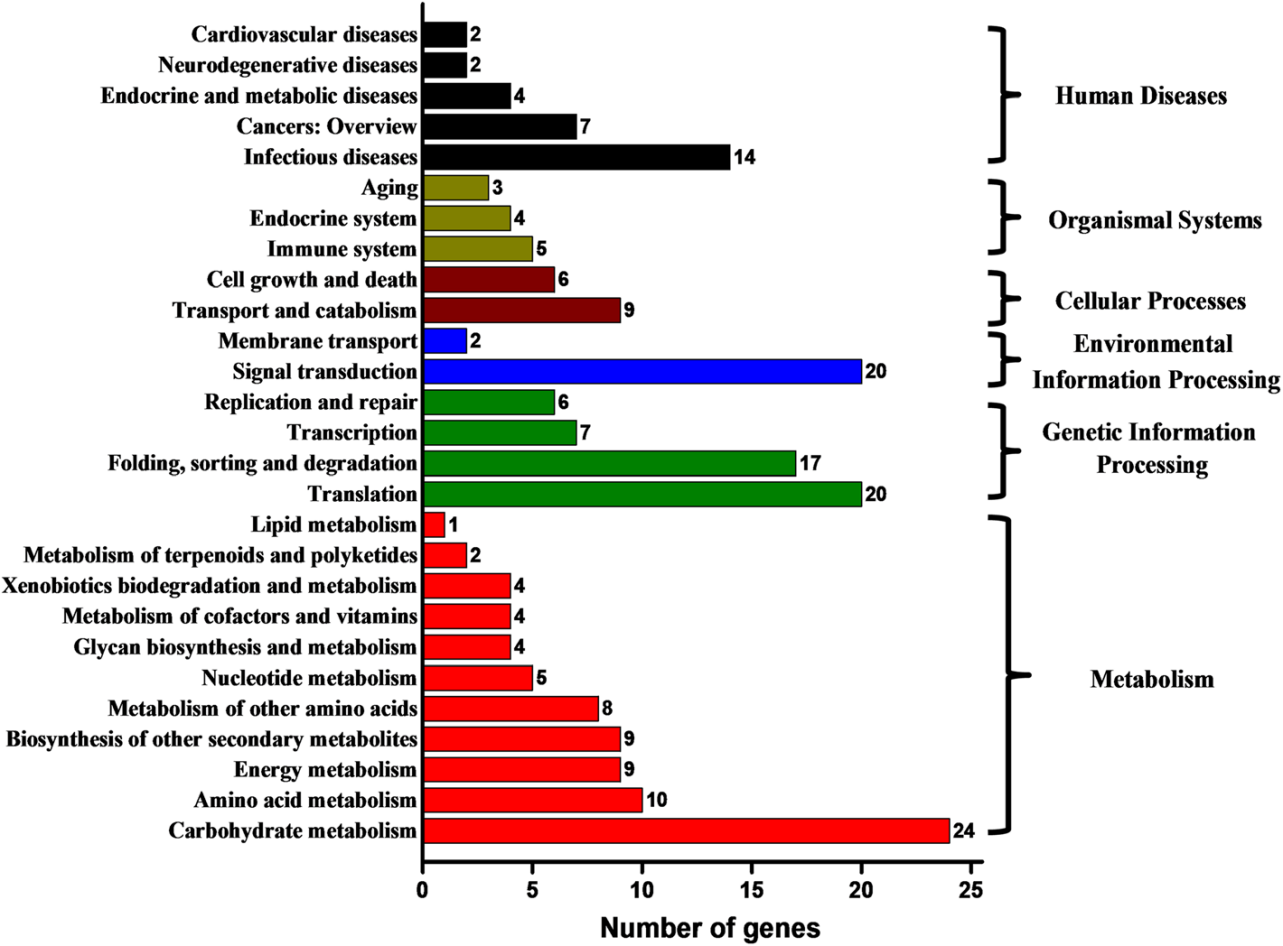
KEGG classification of predicted rice target genes of Southern rice black-streaked dwarf virus-derived siRNAs

Furthermore, we conducted KEGG classification for the predicted target genes of vsiRNAs. In total, 129 genes were classified into six classes – metabolism, genetic information processing, environmental information processing, cellular processes, organismal systems, and human diseases – and then further assigned to 27 KEGG pathways (Fig. 4). It showed that the five most involved pathways were “carbohydrate metabolism”, “translation”, “signal transduction”, “folding, sorting and degradation” and “infectious diseases”, which represented 74% of the annotated genes.

**Fig. 5:**
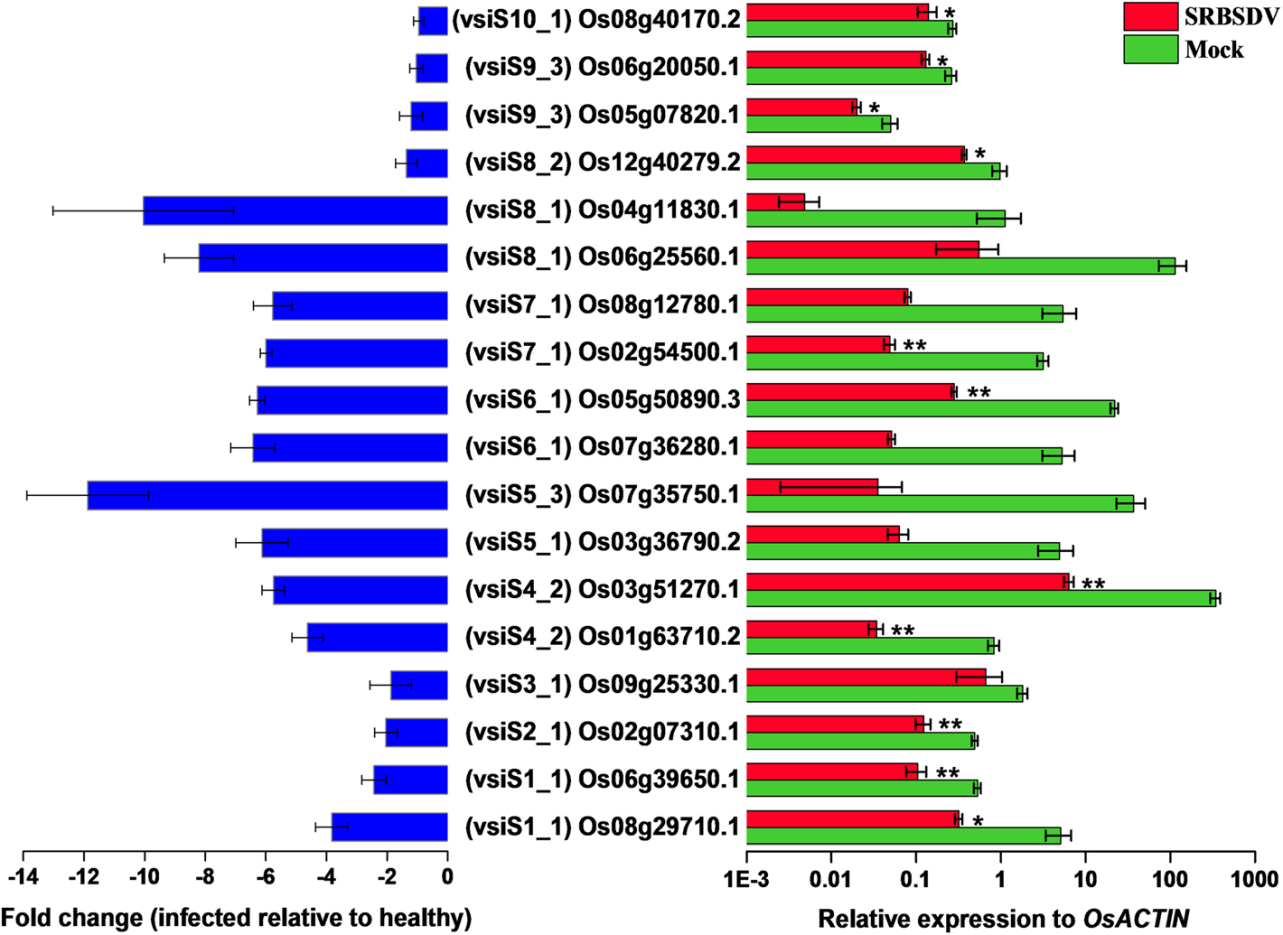
Rice targets putatively down regulated by the most abundant siRNAs from each viral genomic segment. The single and double asterisks indicate significance levels of 0.05 and 0.01 respectively

To check whether the predicted target genes were down regulated by vsiRNAs, we carried out RT-qPCR confirmation for 18 selected rice genes, which putatively were targeted by the 12 vsiRNAs of high abundance (>100 reads, except vsiRS2_1) coming from every genomic segments (Table 2). The result showed that the all the 18 genes were down regulated (fold change = −0.96 ~ −11.88) and 11 of them had significantly decreased expression levels, in the SRBSDV-infected sample (28 dpi) compared to the uninfected control (Fig. 5). However, all the 18 genes had no differential expression between the infected and virus-free samples at 14 dpi. These results suggested that these highly expressed vsiRNAs can most likely target some certain host genes.

**Table 2:** The abundantly expressed Southern rice black-streaked dwarf virus-derived siRNAs and their predicted targets confirmed by RT-qPCR

vsiRNA No.^a)^	ID of predicted target gene	Expectation score ^b)^	Target Accessibility (UPE) ^b)^	Alignment	Inhibition manner	Putative function of rice target gene
vsiRS1_1	LOC_Os08g29710.1	3.0	14.152	miRNA 20 GCGUCUCACAUAGAGCUAAU 1 .::::: :::::::.:::: Target 1652 UGCAGAUUGUAUCUUGAUUU 1671	Cleavage	galactosyltransferase family protein
	LOC_Os06g39650.1	3.0	19.7	miRNA 20 GCGUCUCACAUAGAGCUAAU 1 .::::.::::: ::.::::. Target 1557 UGCAGGGUGUAGCUUGAUUG 1576	Translation	pentatricopeptide
vsiRS2_1	LOC_Os02g07310.1	2.5	20.512	miRNA 21 UCACAAUAGUAUAAGAUACCU 1 .:: :::::::.::::.:::. Target 2336 GGUAUUAUCAUGUUCUGUGGG 2356	Cleavage	AGO17
vsiRS3_1	LOC_Os09g25330.1	3.0	7.741	miRNA 22 CUAAGAAGUUAA-GACGCAGAUU 1 : :::::.:::: ::::::.::: Target 158 GUUUCUUUAAUUGCUGCGUUUAA 180	Translation	Bric-a-Brac, Tramtrack, Broad Complex BTB domain with non-phototropic hypocotyl 3 NPH3 domain, BTBN19
vsiRS4_2	LOC_Os01g63710.2	3.0	11.886	miRNA 20 UGUCAAACGUAACAGAGCAU 1 :::::::::::::: :: :: Target 1668 ACAGUUUGCAUUGUAUCUUA 1687	Cleavage	Putative DNA replication initiation protein, CDC6
	LOC_Os03g51270.1	3.0	14.253	miRNA 20 UGUCAAACGUAACAGAGCAU 1 :::: :: :::: ::::::: Target 1029 ACAGCUUACAUUUUCUCGUA 1048	Cleavage	F-box domain containing protein, OsFBX108
vsiRS5_1	LOC_Os03g36790.2	3.0	21.375	miRNA 20 GUCUUAACUCAGUGCUCACA 1 ::::::: :::..:::::: Target 204 CAGAAUUCAGUUGCGAGUGC 223	Cleavage	tobamovirus multiplication protein
vsiRS5_3	LOC_Os07g35750.1	2.5	18.456	miRNA 20 CACAACAGACGAACAAGCAC 1 ::::::: ::::::::.::: Target 3702 GUGUUGU-UGCUUGUUUGUG 3720	Cleavage	DUF26 kinases homologous to DUF26 containing loci, TKL_IRAK_DUF26-ld.3
vsiRS6_1	LOC_Os07g36280.1	2.5	16.42	miRNA 22 UUGCUAGUGA-AACAAGAGAGUU 1 :: ::::::: :::::.:::::: Target 1053 AAAGAUCACUGUUGUUUUCUCAA 1075	Cleavage	F-box domain containing protein, OsFBX241
	LOC_Os05g50890.3	3.0	23.237	miRNA 22 UUGCUAGUGAAACAAGAGAGUU 1 ..::.::.:::: :::::::.. Target 319 GGCGGUCGCUUUCUUCUCUCGG 340	Translation	Probable indole-3-acetic acid-amido synthetase, OsGH3.5
vsiRS7_1	LOC_Os02g54500.1	2.5	14.148	miRNA 20 AGGAUGUCUUACAAUAAGUU 1 :: ::::::::::::::: : Target 3812 UCAUACAGAAUGUUAUUCUA 3831	Cleavage	WD40-like, putative, expressed
	LOC_Os08g12780.1	3	17.599	miRNA 21 AAGGAUGUCUUACAAUAAGUU 1 :::::.:::.: :::::::: Target 1935 UUCCUGCAGGAAAUUAUUCAA 1955	Translation	chloroplast envelope membrane protein
vsiRS8_1	LOC_Os06g25560.1	2.5	13.472	miRNA 20 CAGCUACAUCUAAGAUCACU 1 :.::::: ::::::::::: Target 454 CUUGAUGUUGAUUCUAGUGA 473	Cleavage	retrotransposon protein
	LOC_Os04g11830.1	3.0	16.405	miRNA 20 CAGCUACAUCUAAGAUCACU 1 :: :::::.:: :.:::::: Target 1708 GUGGAUGUGGAAUUUAGUGA 1727	Translation	TCP family transcription factor
vsiRS8_2	LOC_Os12g40279.2	2.5	17.285	miRNA 20 AGGAUGAGCACGGUAACGCU 1 ::.::::: :::.::::::. Target 3774 UCUUACUCUUGCUAUUGCGG 3793	Cleavage	protein kinase domain containing protein
vsiRS9_3	LOC_Os06g20050.1	3.0	18.094	miRNA 20 CGGUUCAGGUUAACUAGACA 1 ::::::.:::::::: ::: Target 2505 UCCAAGUUCAAUUGAUAUGU 2524	Cleavage	leucine-rich repeat receptor protein kinase EXS precursor
vsiRS9_3	LOC_Os05g07820.1	3.0	17.222	miRNA 20 CGGUUCAGGUUAACUAGACA 1 :.:.:::::: ::::::::: Target 586 GUCGAGUCCA-UUGAUCUGU 604	Translation	NBS type disease resistance protein
vsiRS10_1	LOC_Os08g40170.2	3.0	19.095	miRNA 21 UCGCGGGCUAGAAUAGGUAUU 1 ::.:.:.::::::.:::::.. Target 723 AGUGUCUGAUCUUGUCCAUGG 743	Cleavage	cyclin-dependent kinase B2-1

a) vsiRS2_1: 5’-UCCAUAGAAUAUGAUAACACU-3’, (+) 856–876 nt, GC% = 28.6%, 58 reads. See Table 1 for the information of other vsiRNAs. b) Calculated using psRNATarget (http://plantgrn.noble.org/psRNATarget/) [48]

### Differential expression analysis of several RNA silencing components

**Fig. 6:**
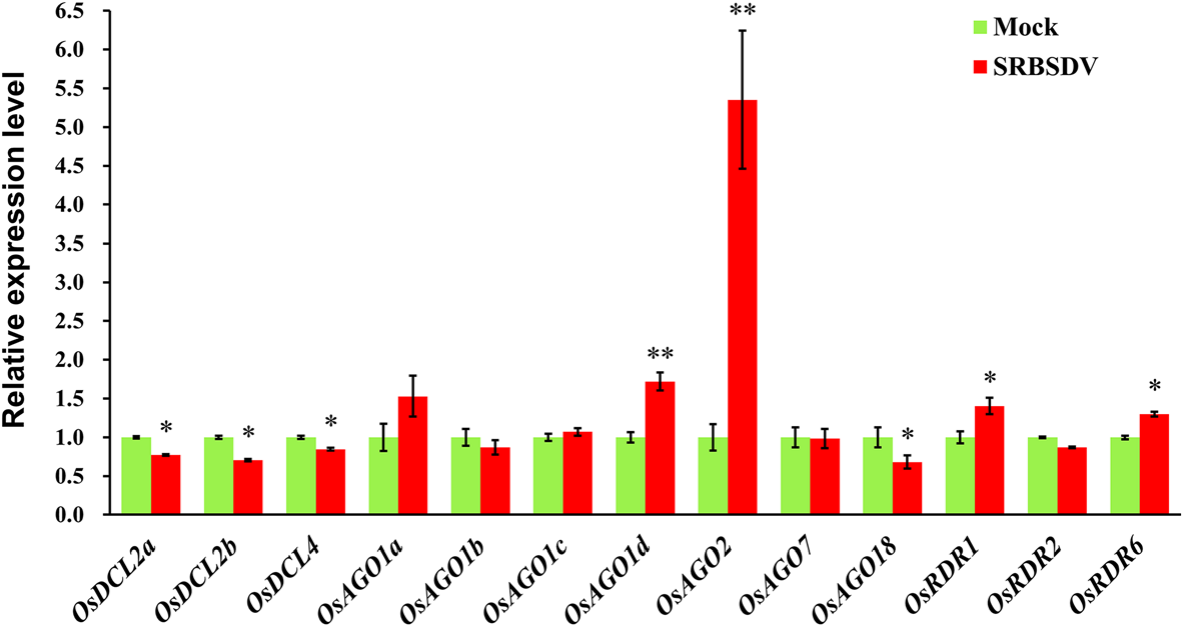
Expression levels of several rice RNA silencing component genes in infected host relative to the uninfected control. The single and double asterisks indicate significance levels of 0.05 and 0.01 respectively

To investigate the influence of SRBSDV infection on the RNA silencing pathways in rice, we compared the expression levels of some representative silencing components between the SRBSDV-infected and mock-inoculated samples. The results revealed that *OsDCL2a*, *OsDCL2b*, *OsDCL4*, and *OsAGO18* were significantly down regulated (among which *OsDCL2a* was predicted as a target of vsiRS8_16 in this study, as aforementioned), while *OsAGO1d*, *OsAGO2*, *OsRDR1* and *OsRDR6* significantly up regulated, in the infected sample compared with the no infection control (Fig. 6). Notably, the abundance of *OsAGO2* became 5.35 fold higher in the infected sample, suggesting its key role implicated in the host’s response to and/or defense against the virus infection.

## Discussion

With the advantage of next-generation sequencing technology, vsiRNA populations have been characterized from a variety of origins, including animal and fungal viruses, and many plant viruses of ssRNA or DNA genomes [8, 9, 68–71], while the characteristics of dsRNA plant virus-derived siRNAs remain relatively less studied. Li et al. profiled the vsiRNAs in *Laodelphax striatellus* infected with RBSDV, the species most closely related to SRBSDV, and reported that they were predominantly 21 and 22 nt long, equally derived from both strand orientations, and frequently produced from hotspots located in 5’- or 3’-terminal regions of viral genomic segments [72]. A most recent study focusing on WBPH miRNA profile also reported the biogenesis of SRBSDV siRNAs without strand favoritism in the viruliferous insect, but the authors did not provide further information about the vsiRNAs [45]. In this study, we expanded the knowledge of dsRNA plant virus-derived siRNAs by presenting the first detailed profile of SRBSDV siRNAs in rice at early and later periods of infection (i.e. 14 and 28 dpi). Interestingly, we found that the regions covering up to 20 ~ 25% of the length of SRBSDV S1 ~ S3 did not produce vsiRNAs. Since a same given virus in different hosts may generate diverse vsiRNA profiles in terms of polarity in strand origins, population constitution, distribution hotspot and pattern in the viral genome, etc. [73], further study will reveal the diversity of SRBSDV siRNA profiles from different hosts like rice, maize and WBPH. Similar to RBSDV- or RSV-derived siRNAs, SRBSDV siRNA population underwent temporal alterations in constitution and abundance (Fig. 1E), and it was likely that increased expression of viral RNA provided more substrates for vsiRNA production [72, 73]. This indicated that the virus triggered the host’s RNA silencing machinery and overcame it to establish successful infection.

vsiRNAs are known to be the core component of RNA silencing that direct host AGOs to cleave viral mRNAs at the complementary sites [69]. In general, vsiRNAs are abundantly produced and heterogeneously distributed throughout the viral genome with a number of biogenesis hotspots [8, 9, 15, 29, 30, 70, 73]. Researchers have found that the efficiencies of vsiRNAs in targeting viral RNA are not correlated with whether they come from hotspot regions [9], suggesting that hotspot vsiRNAs have some biological functions other than targeting viral genes. Evidences have demonstrated that vsiRNAs can regulate host genes by exploiting the RNA silencing mechanism, and therefore enhance their fitness and influence host pathogenesis. The siRNAs derived from the satellite RNA of cucumber mosaic virus-Y (genus *Cucumovirus*, family *Bromoviridae*) was found to down regulate the key gene (*CHLI*) involved in chlorophyll biosynthesis, accounting for the yellowing symptom in *Nicotiana benthamiana* [12]. Peach latent mosaic viroid produces two siRNAs that target the chloroplastic heat shock protein 90 gene in peach to induce host albinism and potentially create an improved host environment for infection [27]. A siRNA derived from potato spindle tuber viroid may silence two callose synthase genes, greatly affecting the disease severity and viroid accumulation [28]. Also, the vsiRNAs present in the maize infected with sugarcane mosaic virus (SCMV, genus *Potyvirus*, family *Potyviridae*) and/or maize chlorotic mottle virus (MCMV, genus *Machlomovirus*, family *Tombusviridae*) were predicted to regulated multiple host genes [29, 30]. In our study, we identified a bunch of SRBSDV siRNAs potentially targeting plentiful rice genes involved in various processes and pathways (Figs. 3 and 4), especially the genes related to disease resistance/stress response, pathogenesis, and developmental processes of the host. We then confirmed the down regulation of a part of predicted rice target genes in infected samples via RT-qPCR (Table 2, Fig. 5). Our results suggested that SRBSDV infection might generally reduce the host’s tolerance to multiple kinds of other pathogens or abiotic stresses and thus aggravate the severity and epidemic of diseases in rice growing areas. In natural conditions, coinfection of SRBSDV and another rice reovirus, rice ragged stunt virus (genus *Oryzavirus*, family *Reoviridae*), has been found in southern China, and their synergism enhances symptom development, virus accumulation, and vector acquisition and transmission of both the viruses [74, 75]. The synergistic co-infection of SCMV and MCMV could increase accumulation of both viruses-derived siRNAs in maize [30]. Whether similar phenomenon occurs in SRBSDV and RRSV co-infected rice and the role of vsiRNAs implicated in their synergism deserve to be uncovered. We also found that vsiRNAs might down regulate several chloroplast-related genes, the pollen development-related gene *AGO17*, and many carbohydrate metabolism-related genes (Table 2, Figs. 4 and 5), suggesting the potential roles of vsiRNAs in development of the leaf symptoms, barren grains and dwarfism caused by SRBSDV [31, 32].

To address the interactions of SRBSDV and rice RNA silencing pathway, we examined the differential expression of several representative silencing component genes crucial to plant antiviral defense. Down regulation of *OsDCL2* and *OsDCL4* in SRBSDV-infected rice plants may influence the production of 21- and 22-nt vsiRNAs and thus weaken host defense, allowing viruses to establish successful infection. *OsDCL4* is known as the SHOOT ORGANIZATION 1 gene (*SHO1*), and studies have discovered that mutations of *OsDCL4* and *OsRDR6* can induce abnormal expression of *Auxin Response Factors* (*ARFs*) and result in many growth abnormities including defects in spikes and lateral organs [76–78]. The down regulation of *OsDCL4* in SRBSDV-infected rice might partially account for the symptoms of small spikes and poorly-developed roots [31, 32]. Many VSRs can block biogenesis of vsiRNAs by interacting with plant DCLs [79]; for example, OsDCL4 is suppressed by RYMV P1 [23]. It is yet to validate whether SRBSDV P6 interacts with the two OsDCLs. Plant AGO1 and AGO2 participate in host defense against many species of RNA viruses and can be suppressed by VSRs [79, 80]. In rice resistance against RDV and RSV, OsAGO18 protects OsAGO1 from being silenced by miR168, conferring a broad-spectrum antivirus resistance [24]. In our study, SRBSDV infection induced up regulation of *OsAGO1* and *OsAGO2* but down regulation of *OsAGO18*, and in particular, *OsAGO2* was drastically up regulated (Fig. 6), suggesting facilitated host antiviral defense. The dramatically activated AGO2 might not only act in vsiRNA binding, but also have additional biological functions, such as participating in miRNA-mediated host gene regulation responding to SRBSDV infection [42] or counterbalancing the suppression of AGO2 by AGO1 via miRNA pathways [80]. Whether the down regulated *OsAGO17* in SRBSDV infected plant affected RNA silencing-directed antiviral defense is unclear. OsRDR6 plays role in defense against RSV and RDV [25, 26], and it is crucial to biogenesis of 21-nt and 24-nt small RNAs and required for normal development of spikelets [77, 78]. In our study, *OsRDR1* and *OsRDR6* were up regulated in infected rice, implying their roles in host response to SRBSDV infection. In general, our results suggested the complicated interactions between the virus and rice RNA silencing pathway, which likely are modulated by the intertwined effects of multiple silencing components.

## Conclusions

In this study, we profiled the vsiRNAs in SRBSDV-infected rice plants, and explored their potential roles in targeting a series of rice genes related to host defense, pathogenesis and symptomology. We also found that SRBSDV infection could affect the expression of several rice RNA silencing pathway components. The results obtained in this study provide an understanding of SRBSDV-derived siRNAs and an insight into the implications of RNA silencing pathway in reovirus-rice interaction.

## Additional files

Additional file 1: Table S1. List of the RT-qPCR primers used in this study. (XLSX 10 kb)
Additional file 2: Table S2. List of the SRBSDV-derived siRNAs identified by deep sequencing from infected rice samples at 14 dpi. (XLSX 70 kb)
Additional file 3: Table S3. List of the SRBSDV-derived siRNAs identified by deep sequencing from infected rice samples at 28 dpi. (XLSX 1015 kb)
Additional file 4: Table S4. List of the SRBSDV-derived siRNAs identified at both 14 dpi and 28 dpi. (XLSX 28 kb)
Additional file 5: Table S5. List of counts of the nucleotides in SRBSDV-derived siRNAs against nt position on the viral genome. (XLSX 433 kb)
Additional file 6: Table S6. List of putative target genes of SRBSDV-derived siRNAs of >40 reads predicted by psRNATarget. (XLSX 78 kb)

## Abbreviations

AGO: Argonaute
DCL: Dicer-like
dpi: Days post inoculation
HR: Hypersensitive response
RDR: RNA-dependent RNA polymerase
RISC: RNA-induced silencing complex
RT-qPCR: Reverse transcription-quantitative PCR
SRBSDV: *Southern rice black-streaked dwarf virus*
vsiRNAs: Virus-derived siRNAs
VSR: Viral suppressor of RNA silencing
